# Maintenance of iodine intake

**DOI:** 10.1186/1756-6614-6-S2-A52

**Published:** 2013-04-05

**Authors:** Peter PA Smyth, Robert Burns, Michael Casey, Ru Jin Huang, Thorsten Hoffman, Colin O’Dowd, Harald Berresheim, Colm O’Herlihy

**Affiliations:** 1 University College Dublin and National University of Ireland, Galway, Ireland; 2 Johannes Gutenberg University of Mainz, Germany

## 

Dietary iodine status is routinely assessed by measuring urinary iodine excretion (UI). In most European countries iodine intake is maintained at WHO recommended levels by iodisation of table salt [1]. Exceptions to this practice include Ireland and the UK where only 5% (approximately) of table salt sold is iodine supplemented. However despite the finding of relatively low median UI values in study populations in both Ireland and the UK [2-4] there is little evidence of an increased prevalence of hypothyroidism, overt or subclinical, of non autoimmune pathogenesis [5-8]. In this communication studies on iodine status in the Irish population over the years 1988-2007 are reviewed, as are investigations into how in the absence of salt iodisation, factors such as proximity to the sea or placental iodide transport/storage have a role in providing sufficient iodine to maintain euthyroid status in the study population.

Subjects were not selected on a systematic basis but were a combination of available findings from different Irish populations studied over the years specified. Although most study groups were comprised of adult females, where these were not available, findings from female schoolchildren were assessed. Median values for UI in study populations ranged from approximately 50-140 µg/l. In the absence of iodised salt availability, milk and dairy products constitute a major iodine source but their content shows seasonal variation with a higher iodine content when cattle in winter housing are fed dietary supplements including iodine [9,10]. Thus UI values were lower during the summer months (April to September) and higher in Winter. The low values in Irish subjects were supported by recent findings in a study of UK female schoolchildren [4] where a median UI value of 80 µg/l corresponded to the most recent Irish value of 79 µg/l [11]. Interestingly the lowest regional value in the UK study came from Northern Ireland where Belfast children had a median UI of 62 µg/l with 30% having values < 50 µg/l. Despite the relatively low UI values obtained in the Irish study populations, findings for neonatal TSH assessed over the years 1995-2006 did not exceed 3%, a cut off point indicative of iodine deficiency. However a small but significant trend to higher TSH, within the reference range, was observed [6].

In the absence of iodine supplementation of table salt, dietary iodine intake is entirely opportunistic. Consumption of milk and dairy products obviously plays a part but this communication reports on the investigation of possible other modes of iodine intake aimed at establishing if living near the sea in a seaweed abundant environment, and therefore exposed to gaseous I2 ingestion by respiration, may confer advantages in terms of iodine intake. Also, as adequate iodine nutrition for the foetus depends not only on maternal iodine supply, but also on the ability of the placenta to successfully transport iodide to the foetal thyroid for use in thyroid hormone biosynthesis, it is proposed to report on placental uptake and possible storage of iodine as a means of maintaining adequate iodine intake in utero.

Ireland has traditionally been regarded as an area of borderline iodine deficiency which might not be expected on an island where few live more than 200Km from the sea. However as has become apparent in recent times, availability of iodine in the diet is dependent on many factors, of which consumption of seafoods is but one. Seaweed is the major source of iodine and for this study we utilised for iodine intake studies measurements whose purpose was to determine the concentration of gaseous I2 released by seaweeds and its effect on cloud formation and changes in weather [12,13]. To determine a possible role for seaweed derived atmospheric iodine ingestion in human populations, urine samples were obtained from female schoolchildren and adults living in coastal areas seaweed rich and of low seaweed abundance and in inland areas of Ireland. Atmospheric I2 was greatest over the seaweed mass but crude calculations based on a daily inspiration of 10,000 L of air could only provide a potential increment of ~30 µg/day additional iodine [14]. Most would receive considerably less. Despite this relatively low potential I2 intake, UI in populations living near a seaweed hotspot were much higher than in lower abundance seaweed coastal or inland areas (158, 71 and 58 µg/l, respectively). Higher values (>150 µg/l) were observed in 40.4% of (seaweed rich), 3.6% (lower seaweed), 2.3% (inland)) supporting the hypothesis that iodine intake in coastal regions may be influenced by seaweed abundance rather than proximity to the sea [14].

The other area of study presented is the role of the placenta in iodide transport/storage. How is iodide transported into the placenta? Is iodide transport concentration dependent? Is such transport hormone dependent? Both fresh placental tissue obtained from women undergoing elective prelabour caesarean section at term and primary cultures of trophoblastic cells accumulate radioactive 125I which could be partially blocked by perchlorate inhibition [unpublished observations]. RNA was isolated from placental trophoblasts and real time RT-PCR performed using sodium iodide symporter (NIS) and pendrin (PDS) probes. Trophoblastic cells expressed both NIS and PDS. 125I uptake in primary cultures from placental tissues was enhanced by individual pregnancy related hormones, particularly hCG and oxytocin, with synergism between hormone combinations. These incremental responses were mirrored by increased expression of NIS but not PDS when measured by Real time PCR suggesting that the increased iodide uptake was solely due to increased NIS expression. Measurement of placental tissue iodine content (mean 40ng/g tissue), while not approaching thyroid levels (~ 1,000 ng/G tissue), is significantly greater than that of other non-thyroidal tissues and appears to be directly proportional to iodine intake as determined by UI [11,15].

## Conclusions

The above findings demonstrate that in the absence of an iodine supplementation through iodisation of salt or otherwise, the study population appears to be only borderline iodine deficient. Even this apparent deficiency depends on the applicability to the definition of iodine deficiency of the WHO recommended UI cut off point (Median UI 100 µg/l) as it has been suggested that using the Recommended Daily Allowance (RDA) of the Institute of Medicine (95 µg) would lower this cut off point from 100 µg/l to 63 µg/l [16].

Investigating alternative pathways of iodine intake such as gaseous I2 ingestion or placental iodide transport in utero may help to explain maintenance of adequate iodine intake. However calculation of the potential exposure to gaseous I2 ingestion in children residing in a seaweed abundant coastal area is difficult to reconcile with the relatively high UI values observed. On the basis of measured atmospheric gaseous I2, extremely preferential I2 absorption would be required to make a significant contribution to daily iodine intake. Although living near the sea may not in itself be sufficient to maintain satisfactory iodine intake, findings in this study do not exclude the possibility of a significant role for iodine inhalation in influencing iodine status.

Iodine (125I) uptake in primary cultures from placental tissues was enhanced by individual pregnancy related hormones, particularly hCG and oxytocin with synergism between hormone combinations. These incremental responses were mirrored by increased expression of placental NIS. As only up to 30% of 125I uptake by placental cells was blocked by perchlorate, it is likely that passive diffusion as well as active transport is involved. However iodide uptake does appear to involve placental storage as placental tissue had a significantly higher iodide content than other non-thyroid tissues. Also it has been demonstrated that maternal urinary iodine (UI) excretion in the immediate antenatal and early postpartum periods showed a precipitous fall in median values from 93 µg/l antenatally to 36 µg/l at delivery which could be possibly explained by placental loss [17].

It therefore appears that iodide uptake and transport from other than conventional dietary sources may assist in maintaining normal thyroid function even dietary intake is apparently deficient.
